# Life course, genetic, and neuropathological associations with brain age in the 1946 British Birth Cohort: a population-based study

**DOI:** 10.1016/S2666-7568(22)00167-2

**Published:** 2022-09

**Authors:** Aaron Z Wagen, William Coath, Ashvini Keshavan, Sarah-Naomi James, Thomas D Parker, Christopher A Lane, Sarah M Buchanan, Sarah E Keuss, Mathew Storey, Kirsty Lu, Amy Macdougall, Heidi Murray-Smith, Tamar Freiberger, David M Cash, Ian B Malone, Josephine Barnes, Carole H Sudre, Andrew Wong, Ivanna M Pavisic, Rebecca Street, Sebastian J Crutch, Valentina Escott-Price, Ganna Leonenko, Henrik Zetterberg, Henrietta Wellington, Amanda Heslegrave, Frederik Barkhof, Marcus Richards, Nick C Fox, James H Cole, Jonathan M Schott

**Affiliations:** a Dementia Research Centre, University College London Queen Square Institute of Neurology, London, UK; b Dementia Research Institute, University College London Queen Square Institute of Neurology, London, UK; c Department of Neurodegenerative Disease, University College London Queen Square Institute of Neurology, London, UK; d Genetics and Genomic Medicine, Great Ormond Street Institute of Child Health, University College London, London, UK; e Neurodegeneration Biology Laboratory, The Francis Crick Institute, London, UK; f Department of Brain Sciences, Imperial College London, London, UK; g UK Dementia Research Institute Centre for Care Research and Technology, Imperial College London, London, UK; h Medical Research Council Unit for Lifelong Health and Ageing, University College London, London, UK; i Department of Medical Statistics, London School of Hygiene & Tropical Medicine, London, UK; j Dementia Research Institute, Cardiff University, Cardiff, UK; k Division of Neuroscience and Mental Health, Cardiff University, Cardiff, UK; l Department of Psychiatry and Neurochemistry, Institute of Neuroscience and Physiology, the Sahlgrenska Academy at the University of Gothenburg, Mölndal, Sweden; m Clinical Neurochemistry Laboratory, Sahlgrenska University Hospital, Mölndal, Sweden; n Department of Computer Science, Centre for Medical Imaging Computing, University College London, London, UK; o Department of Radiology and Nuclear Medicine, Amsterdam University Medical Centre, Vrije Universiteit, Amsterdam, Netherlands; p School of Biomedical Engineering and Imaging Sciences, King's College London, London, UK

## Abstract

**Background:**

A neuroimaging-based biomarker termed the brain age is thought to reflect variability in the brain's ageing process and predict longevity. Using Insight 46, a unique narrow-age birth cohort, we aimed to examine potential drivers and correlates of brain age.

**Methods:**

Participants, born in a single week in 1946 in mainland Britain, have had 24 prospective waves of data collection to date, including MRI and amyloid PET imaging at approximately 70 years old. Using MRI data from a previously defined selection of this cohort, we derived brain-predicted age from an established machine-learning model (trained on 2001 healthy adults aged 18–90 years); subtracting this from chronological age (at time of assessment) gave the brain-predicted age difference (brain-PAD). We tested associations with data from early life, midlife, and late life, as well as rates of MRI-derived brain atrophy.

**Findings:**

Between May 28, 2015, and Jan 10, 2018, 502 individuals were assessed as part of Insight 46. We included 456 participants (225 female), with a mean chronological age of 70·7 years (SD 0·7; range 69·2 to 71·9). The mean brain-predicted age was 67·9 years (8·2, 46·3 to 94·3). Female sex was associated with a 5·4-year (95% CI 4·1 to 6·8) younger brain-PAD than male sex. An increase in brain-PAD was associated with increased cardiovascular risk at age 36 years (β=2·3 [95% CI 1·5 to 3·0]) and 69 years (β=2·6 [1·9 to 3·3]); increased cerebrovascular disease burden (1·9 [1·3 to 2·6]); lower cognitive performance (–1·3 [–2·4 to –0·2]); and increased serum neurofilament light concentration (1·2 [0·6 to 1·9]). Higher brain-PAD was associated with future hippocampal atrophy over the subsequent 2 years (0·003 mL/year [0·000 to 0·006] per 5-year increment in brain-PAD). Early-life factors did not relate to brain-PAD. Combining 12 metrics in a hierarchical partitioning model explained 33% of the variance in brain-PAD.

**Interpretation:**

Brain-PAD was associated with cardiovascular risk, and imaging and biochemical markers of neurodegeneration. These findings support brain-PAD as an integrative summary metric of brain health, reflecting multiple contributions to pathological brain ageing, and which might have prognostic utility.

**Funding:**

Alzheimer's Research UK, Medical Research Council Dementia Platforms UK, Selfridges Group Foundation, Wolfson Foundation, Wellcome Trust, Brain Research UK, Alzheimer's Association.

## Introduction

Ageing is associated with substantial interindividual effects on function, morbidity, and mortality. A reliable cross-sectional metric that can quantify this variability—a measure of biological age—would be valuable both for clinical practice and research into longevity and ageing health. This metric could facilitate the monitoring of age-related changes beyond that captured by disease specific risk factors—ie, by incorporating mechanisms of decline due to both disease and typical ageing.^1^ Likewise, the metric could help to detect people who are ageing more rapidly than expected, before the onset of clinical manifestations,^2^ alongside being able to detect traits related to delayed ageing, cognitive maintenance, and longevity.

The concept of brain age examines biological ageing from a neuroanatomical perspective.^3^, ^4^ Using machine learning to compare an individual's structural magnetic resonance image (T1-weighted MRI) with a large reference dataset of healthy brains allows prediction of a biological brain age. This brain age measure can be subtracted from chronological age to determine the brain-predicted age difference (brain-PAD). Over and above associations with structural brain volumes,^5^, ^6^ brain-PAD has been shown to predict 8-year mortality of 70-year-old individuals,^7^ and to be associated with physical function, risk of developing dementia,^5^, ^8^ and neuropsychiatric diseases including Alzheimer's disease, multiple sclerosis, and depression.^3^, ^9^, ^10^ Mid-life brain age has been associated with reduced cognitive function and early signs of cognitive decline from childhood until midlife.^11^

**Research in context**

**Evidence before this study**

We searched PubMed for studies published in English from inception up to Aug 9, 2021, using the key terms “brain-age”, “brain predicted age”, and “brain-predicted age difference”, in combination with “biological age” and “neurodegeneration”. Systematic reviews showed associations between brain-predicated age difference and genetic and fluid biomarkers of age-related diseases including Alzheimer's disease, as well as mid-life risk for later cognitive dysfunction, and risk of 8-year mortality of 70-year-old individuals.

**Added value of this study**

This work extends previous research by applying brain age to a unique birth cohort study, ongoing for 72 years, with the rich life-course data showing that brain-predicted age difference (brain-PAD) associates with middle and later life metrics such as cardiovascular risk, rather than early-life and demographic measures. In addition, the study explored novel modalities, showing associations between brain-PAD and the blood-based biomarker serum neurofilament light, and the association of brain-PAD to subsequent regional brain atrophy over 2 years.

**Implications of all the available evidence**

Brain-PAD provides a single summary metric integrating brain decline due to diseases and normal ageing and it relates to a neurochemical marker of neurodegeneration. As a cross-sectional marker, brain-PAD might help to identify people at risk of future cognitive decline and poorer brain-health-related outcomes.

Many studies investigating biological variability in ageing are limited by the variability of chronological age among participants, a dependence on retrospective data collection, and heterogeneity in image acquisition and processing. The Medical Research Council National Survey of Health and Development (NSHD), also known as the 1946 British Birth Cohort, is the world's longest continuously running birth cohort and provides the opportunity to assess relationships among biological contributors to ageing. Alongside effectively controlling for chronological age, members of this cohort have been extensively studied since birth, with 24 prospective waves of data collection over the life course. Insight 46 is a substudy of the NSHD where, at the age of approximately 70 years, 502 members of the cohort were recruited to a longitudinal study incorporating amyloid β-PET and multimodal MRI (PET-MRI) on a single scanner, detailed cognitive assessments, physical examination, and measurement of blood-based biomarkers.^12^

Here we applied a well-established brain age model to derive brain-PAD from structural imaging in Insight 46 participants. Using life course data and contemporaneous measures of cognition and brain pathologies, we aimed to explore associations between brain-PAD and factors hypothesised to influence brain ageing, including markers of Alzheimer's disease and cerebrovascular disease. Specifically, we investigated how brain-PAD relates to five outcomes: childhood, midlife, and late-life-course measures; imaging-based markers of neurodegeneration, including Alzheimer's disease and cerebrovascular diseases; cognitive performance; blood-based biomarkers; and subsequent rates of brain atrophy.

## Methods

### Study design and participants

**Figure 1:**
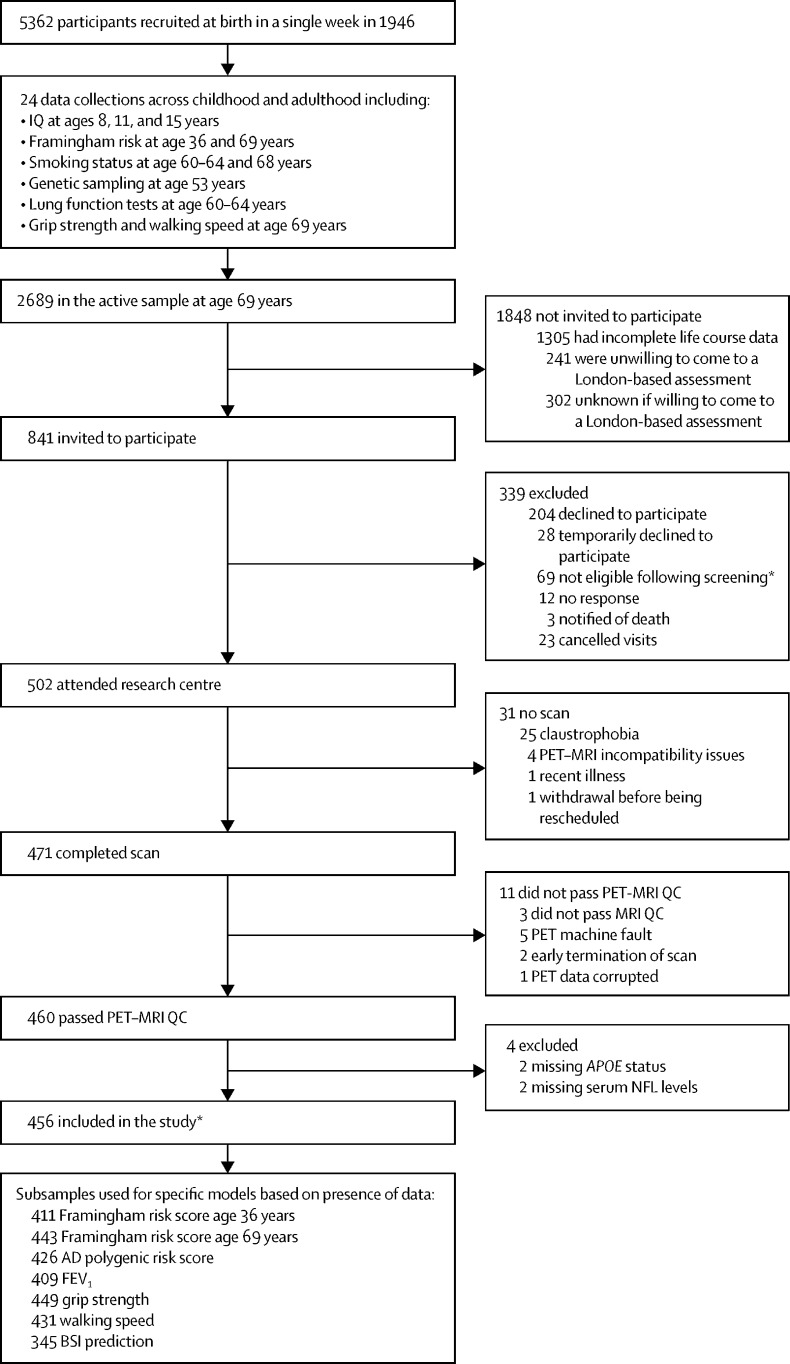
Study profile AD=Alzheimer's disease. BSI=boundary shift integral. FEV_1_=forced expiratory volume. IQ=intelligence quotient. NFL=neurofilament light. QC=quality control. *Included 41 participants with major brain disorders: dementia (n=3), psychiatric disorder requiring antipsychotic treatment or electroconvulsive shock therapy (n=4), radiological evidence of possible brain malignancy (n=1), epilepsy (n=6), hepatic encephalopathy (n=1), clinical diagnosis or radiological features of multiple sclerosis (n=3), myotonic dystrophy (n=1), Parkinson's disease (n=2), Parkinson's disease and epilepsy (n=1), clinical diagnosis of stroke or radiological evidence of cortical stroke (n=17), and traumatic brain injury or major neurosurgery (n=2).

5362 participants were born in mainland Britain in the same week in March, 1946, and were recruited to the NSHD at birth. This cohort has undergone 24 assessments since birth as part of the NSHD. 502 NSHD participants were recruited to the Insight 46 study (figure 1).^12^, ^13^ Ethics approval was obtained for the wider NSHD, and for Insight 46 by the National Research Ethics Service Committee (14/LO/1173). All participants provided written consent.

### Childhood metrics and demographics

Childhood cognitive ability was assessed at age 8 years by combining four tests of verbal and non-verbal ability into a Z score standardised over the full NSHD cohort.^14^ If data were missing, the equivalent score was taken from age 11 years (or age 15 years if both metrics were missing). Adult socioeconomic position was defined as non-manual or manual, on the basis of the occupation between the ages of 15 and 53 years, according to the UK Registrar General's Classification of Occupations. Educational attainment was defined as the highest qualification by age 26 years, divided into three categories: none attempted; vocational or GCSE; and A level or higher. Smoking status was assessed via questionnaire at age 68 years (or, if that data were missing, at age 60–64 years) and divided into three categories: never smoked, ex-smoker, or current smoker.

### Midlife factors

A clinic-based Framingham Heart Study Cardiovascular Risk Score (FRS) was derived at multiple times during the life course.^15^ The FRS incorporates age, sex, systolic blood pressure, antihypertensive medication use, BMI, diabetes history, and smoking status to estimate a 10-year risk of a major cardiac event. Previous studies of the Insight 46 cohort have shown that FRS at age 36 years has the greatest effect on brain volume and white matter hyperintensity (WMH) volume in later life.^15^ Given this finding, we studied FRS at age 36 years and concurrently with the imaging assessments, to capture a broader range of vascular risk factors.

### Contemporaneous factors

All participants had a clinical assessment between May 28, 2015, and Jan 10, 2018, at University College London, UK. Age was defined as age at the time of brain imaging, or, if no scan was undertaken, then age at the time of blood test. Height was measured by a study doctor.

Imaging was performed and analysed as previously described and as detailed in the appendix (p 1), including MRI measures of cross-sectional brain volumes and WMH volume, and direct measures of brain volume change between baseline and the repeat scan 2 years later, assessed using the boundary shift integral.^12^, ^16^ Fibrillar amyloid β was quantified following injection of 370 MBq [^18^F] florbetapir (Avid Radiopharmaceuticals, Philadelphia, PA, USA) amyloid β-PET ligand with generation of a global standardised uptake value ratio (SUVR) using an eroded white matter reference region as previously described.^16^ Amyloid positive status was defined using a Gaussian mixed model using the 99th percentile of the lower (amyloid negative) distribution as a cutoff: equivalent to an SUVR of 0·671, or 17 centiloids. A radiologist assessed the images for major brain disorders.

Brain age processing used Gaussian Processes regression, implemented in the brainageR software package version 1.0,^4^, ^7^ to derive brain-predicted age from T1-weighted MRI scans. This model is highly similar to that used in our previous research,^10^, ^17^, ^18^, ^19^, ^20^, ^21^, ^22^ although implemented in R instead of Matlab.^23^ BrainageR was trained on MRI scans from 2001 healthy adults aged 18–90 years. Raw T1-weighted MRI sequences from Insight 46 participants were passed through the brainageR software, which includes pre-processing with SPM12 segmentation and DARTEL spatial normalisation, before generation of brain-predicted age values from the principal components of normalised maps of grey matter, white matter, and cerebrospinal fluid. Chronological age was then subtracted from brain-predicted age to derive brain-PAD.

*APOE* ɛ4 status (non-carrier *vs* carrier) was measured at age 53 years by genotyping two single nucleotide polymorphisms (SNPs), rs439358 and rs7412. DNA from each participant was extracted by standard methods and genotyped using the NeuroX2 (Infinium NeuroConsortium Array; Illumina, San Diego, USA) and DrugDev genomic arrays (Infinium DrugDev Consortium Array; Illumina). An Alzheimer's disease Polygenic Risk Score was derived from 2864 individuals and 486 137 SNPs from the NeuroX2 platform, and 2851 samples using 476 728 SNPs from the DrugDev platform (appendix p 1). Non-fasted serum samples for blood-based biomarker detection were collected at age 70 years via peripheral venepuncture, and serum neurofilament light (NFL) concentrations were assessed in duplicate using the Simoa immunoassay NF-Light kits (Quanterix; Billerica, MA, USA).

Grip strength was measured in kg at age 69 years using a Jamar Plus + Digital Hand dynamometer (Rolyn Prest, Colorado, USA), taken as the maximum of four attempts. Forced expiratory volume in 1 s (FEV_1_) was assessed at age 60–64 years as the maximum score of at least two values between 0·3 L and 0·9 L, where the difference between the values was less than 0·3 L. Walking speed was assessed at age 69 years as the average time taken from two attempts to walk 10 m.

Adult cognition was assessed using the Preclinical Alzheimer Cognitive Composite Score (PACC) comprising the Mini Mental State Examination (MMSE), Digit-Symbol Substitution test from the Weschler Adult Intelligence Scale-Revised, the Logical Memory IIa from the Wechsler Memory Scale-Revised, and the 12 item Face–Name test.^14^ Z scores for each of these four tests were averaged to derive the PACC. Dementia status was assigned by expert consensus on the basis of clinical history, informant history, and MMSE score of less than 26.

### Statistical analysis

Using brain-PAD values as outcomes, statistical analysis was undertaken in R 4.1.0. Statistical significance was set at p<0·05. Multivariable linear regression was used to assess relationships between all predictors and brain-PAD; where relevant, continuous variables were scaled to Z scores to facilitate comparisons. The models used and metrics included are summarised in the appendix (p 8). Independent models were defined for each of the demographic, life course, imaging, biomarker, cognitive, physical, and cardiovascular risk variables, using brain-PAD as the outcome measure, and the respective variable as a predictor. Models incorporating life course and demographic factors, blood biomarkers, WMH, and amyloid imaging were covaried for sex. Cardiovascular risk models were covaried for socioeconomic status. For variables where we observed the potential for outliers to influence results (serum NFL, FRS at age 36 years, and hippocampal boundary shift integral), robust regression was used. Models assessing whole brain, hippocampal, ventricular, and WMH MRI volumes were covaried for total intracranial volume (TIV) and sex. The PACC model used sex, socioeconomic status, childhood cognition, and educational attainment as covariates, as these have previously been shown to be statistically significant contributors.^14^ Physical metrics were covaried with sex. FEV_1_ was additionally covaried for smoking status and height, and walking speed was covaried for height. Examination of residuals was performed to confirm model fits. Hierarchical partitioning of variance was applied to a linear regression on brain-PAD to assess unique and shared variance associated with 12 predictor variables: age, sex, childhood cognition, socioeconomic status, FRS at ages 36 and 69 years, PACC, amyloid SUVR, serum NFL, TIV, whole brain volume (WBV), and WMH volume. Finally, separate linear regressions were used to assess whether baseline brain-PAD related to subsequent rates of change in whole brain, ventricular, and total hippocampal volume, adjusted for sex and TIV, and in a sensitivity analysis for WBV. These final models included change in volume (mL) as the outcome, scan interval in years as the explanatory variable, and interactions between scan interval and the predictor of interest (ie, baseline brain-PAD) and each covariate. In Insight 46, chronological age at time of assessment is affected by order of participant recruitment; therefore sensitivity analysis was conducted using all relevant models with chronological age included as a covariate.

### Role of the funding source

The funders of the study had no role in study design, data collection, analysis, or interpretation, or writing of the report.

## Results

**Figure 2:**
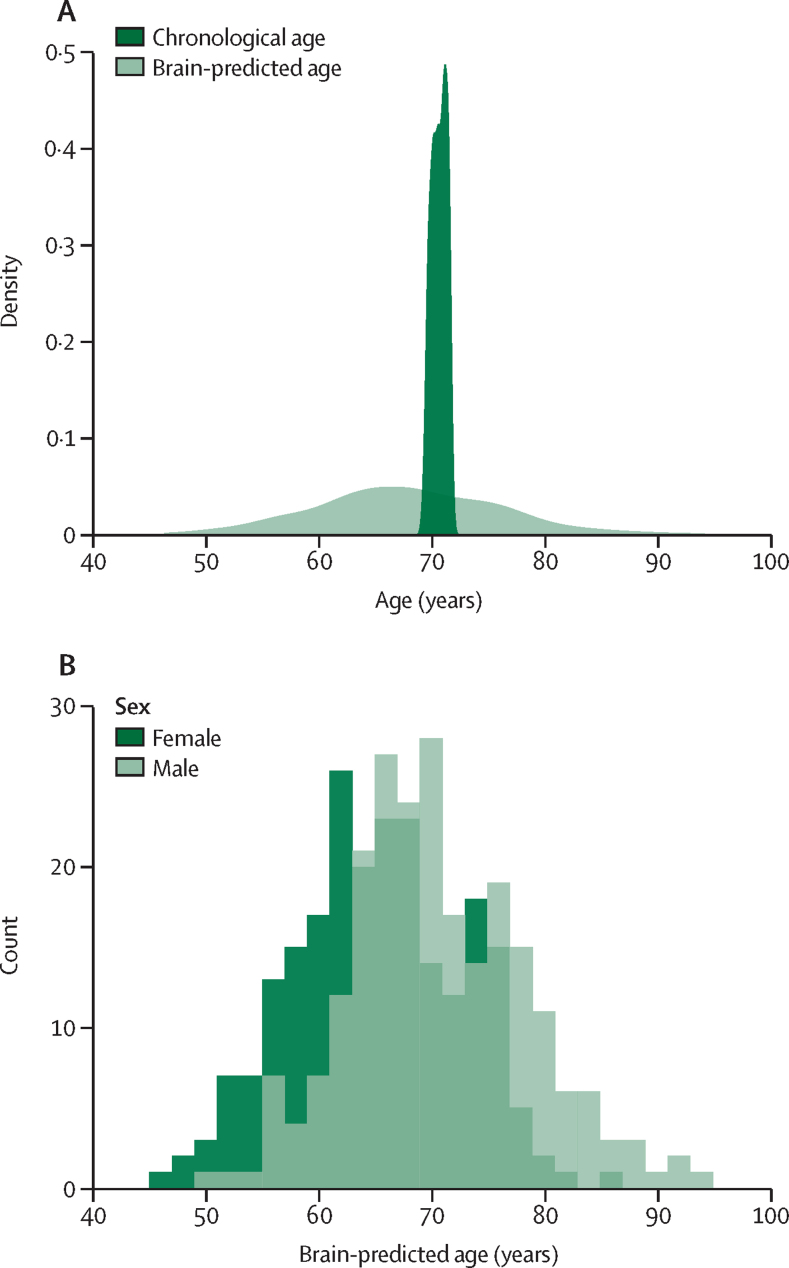
Comparison of brain-predicted age with chronological age and sex (A) Density plot showing the brain-predicted age overlain with chronological age. The brain age algorithm can resolve ages over a 47-year range, even among participants of similar chronological age. The mean brain-predicted age of the participants was 2·77 years younger than chronological age. (B) Histogram showing differences in brain age by sex. Female participants had a mean brain-predicted age 5·5 years younger than male participants.

456 (91%) of 502 participants recruited to Insight 46 were included in the study on the basis of having complete imaging, serum NFL, and *APOE* data (figure 1, table). 415 (91%) of these participants were cognitively typical with no major brain disorder. Subsamples were used for specific analyses where data were missing (figure 1). Comparison of participants included in the study with those excluded (n=46) show no overt differences in age, sex, and demographic metrics (appendix p 4). Despite a very narrow chronological age range of 2·6 years (69·3–71·9 years, SD 0·7), reflecting participants’ age at assessment for Insight 46 (the timeframe required for data collection), brain-predicted age ranged from 46·3 to 94·3 years (SD 8·2 years; figure 2A). Mean brain-predicted age was 67·9 years, 2·8 years younger than the mean chronological age.

**Table:** Participant characteristics

	**Total (n=456)**	**Female (n=225)**	**Male (n=231)**
**Chronological age (years)**			
Range	69·2 to 71·9	69·3 to 71·9	69·2 to 71·8
Mean (SD)	70·7 (0·7)	70·7 (0·7)	70·7 (0·7)
**Brain-predicted age (years)**			
Range	46·3 to 94·3	46·3 to 85·2	50·9 to 94·3
Mean (SD)	67·9 (8·1)	65·2 (7·4)	70·6 (7·9)
**Brain-predicted age difference (years)**			
Range	−24·6 to 22·7	−24·6 to 14·7	−19·9 to 22·7
Mean (SD)	−2·8 (8·0)	−5·5 (7·3)	−0·1 (7·8)
**Socioeconomic status**			
Manual	70 (15%)	30 (13%)	40 (17%)
Non-manual	386 (85%)	195 (87%)	191 (83%)
**Educational attainment**			
None attempted	70 (15%)	37 (16%)	33 (14%)
Secondary education	139 (30%)	82 (36%)	57 (25%)
Higher education	247 (54%)	106 (47%)	141 (61%)
**Childhood cognition, Z score**			
Range	−1·60 to 2·50	−1·59 to 2·47	−1·60 to 2·50
Mean (SD)	0·41 (0·75)	0·44 (0·74)	0·38 (0·75)
**Smoking**			
Never smoked	160 (35%)	86 (38%)	74 (32%)
Ex-smoker	280 (61%)	131 (58%)	149 (65%)
Current smoker	16 (4%)	8 (4%)	8 (3%)
**Major brain disorder**			
None	415 (91%)	207 (92%)	208 (90%)
Present	41 (9%)	18 (8%)	23 (10%)
**Total intracranial volume (mL)**			
Range	1114 to 1939	1114 to 1558	1274 to 1939
Mean (SD)	1431 (133)	1341 (92)	1518 (106)
**Whole brain volume (mL)**			
Range	819 to 1494	819 to 1265	946 to 1494
Mean (SD)	1099 (99)	1045 (82)	1151 (86)
**Ventricular volume (mL)**			
Range	6·16 to 112·00	6·16 to 82·93	9·33 to 112·00
Mean (SD)	30·94 (16·34)	26·43 (14·70)	35·32 (16·68)
**Hippocampal volume (mL)**			
Range	4·12 to 8·54	4·12 to 7·45	4·83 to 8·54
Mean (SD)	6·26 (0·67)	6·01 (0·59)	6·51 (0·65)
**White matter hyperintensity volume (mL)**			
Range	0·27 to 33·67	0·35 to 32·78	0·27 to 33·67
Mean (SD)	5·21 (5·54)	5·64 (5·90)	4·80 (5·15)
**PACC score (Z score)**			
Range	−3·49 to 1·72	−3·48 to 1·67	−3·49 to 1·72
Mean (SD)	−0·01 (0·74)	0·16 (0·73)	−0·17 (0·71)
**Amyloid status**			
Negative	373 (82%)	188 (84%)	185 (80%)
Positive	83 (18%)	37 (16%)	46 (20%)
**Amyloid SUVR (centiloids)**			
Range	−17·94 to 92·84	−17·94 to 90·74	−17·50 to 92·84
Mean (SD)	7·13 (19·05)	6·10 (18·31)	8·15 (19·73)
**Serum neurofilament light (pg/mL)**			
Range	7·26 to 124·00	7·26 to 121·00	7·39 to 124·00
Mean (SD)	20·74 (12·19)	20·95 (10·78)	20·53 (13·45)
APOE **ɛ4 status**			
Non-carrier	325 (71%)	165 (73%)	160 (69%)
Carrier	131 (29%)	60 (27%)	71 (31%)
**Alzheimer's Polygenic Risk Score (Z score)**			
Participants	426	209	217
Range	−3·15 to 2·75	−2·60 to 2·75	−3·15 to 2·14
Mean (SD)	−0·06 (1·01)	−0·12 (0·98)	−0·01 (1·04)
**Framingham Risk Score age 36 years**			
Participants	411	203	208
Range	0·58 to 11·25	0·58 to 5·29	1·69 to 11·25
Mean (SD)	2·90 (1·74)	1·72 (0·79)	4·05 (1·65)
**Framingham Risk Score age 69 years**			
Participants	443	216	227
Range	2·53 to 68·75	2·53 to 62·95	14·46 to 68·75
Mean (SD)	25·90 (13·45)	16·66 (9·20)	34·69 (10·68)
**Forced expiratory volume (L)**			
Participants	409	208	201
Range	0·37 to 4·84	0·84 to 3·76	0·37 to 4·84
Mean (SD)	2·71 (0·68)	2·26 (0·42)	3·18 (0·56)
**Grip strength (kg)**			
Participants	449	220	229
Range	11·00 to 61·50	11·00 to 43·80	14·80 to 61·50
Mean (SD)	32·99 (10·90)	24·48 (5·59)	41·16 (8·16)
**Walking speed (m/s)**			
Participants	431	209	222
Range	0·57 to 2·22	0·57 to 2·22	0·57 to 1·99
Mean (SD)	1·08 (0·26)	1·06 (0·26)	1·11 (0·26)

PACC=Preclinical Alzheimer's Cognitive Composite Score. SUVR=standardised uptake value ratio.

**Figure 3:**
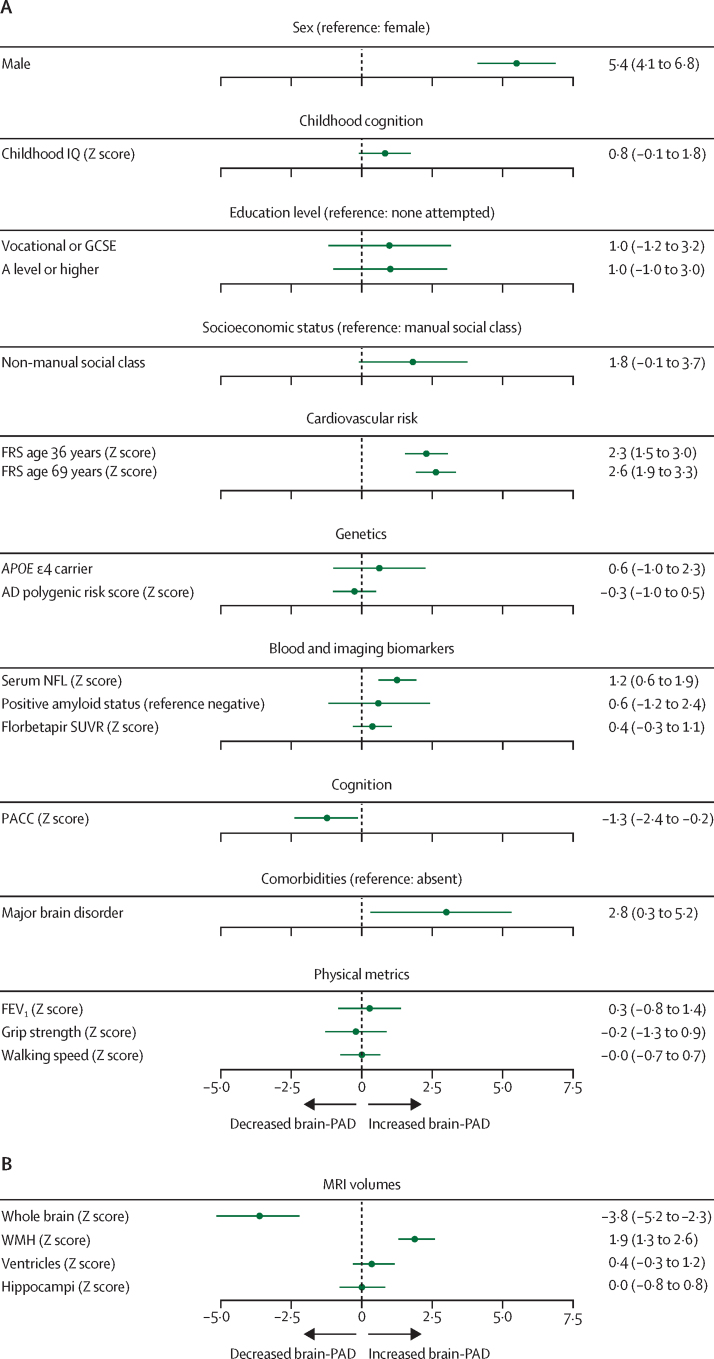
Associations of childhood and midlife, contemporaneous, and imaging factors with brain-PAD Forest plots show results of individual linear regression models of brain-PAD, plotting β coefficients in years (95% CI) with values listed to the right. For continuous variables, a 1 SD increase in the Z score of interest is associated with a β year increase in brain-PAD. (A) Association of demographic, childhood, midlife, and contemporaneous factors with brain-PAD. Models incorporating life course and demographic factors, blood biomarkers, WMH, and amyloid imaging were covaried for chronological age and sex. The PACC model was covaried for chronological age, sex, socioeconomic status, childhood cognition, and education attainment. Cardiovascular risk models were covaried for socioeconomic status. (B) Structural MRI metrics associating with brain-PAD. Models assessing structural MRI factors were covaried for TIV, age, and sex. Serum NFL and Framingham risk at age 36 years were assessed using robust regression. AD=Alzheimer's disease. Brain-PAD=brain-predicted age difference. FEV=forced expiratory volume. FRS=Framingham risk score. IQ=intelligence quotient. NFL=neurofilament light. PACC=Preclinical Alzheimer's Cognitive Composite Score. SUVR=standardised uptake value ratio. TIV=total intracranial volume. WMH=white matter hyperintensity.

The mean brain-predicted age for female participants was 5·4 years (95% CI 4·1–6·8) younger than male participants (65·2 *vs* 70·6 years; figure 2B, figure 3), after adjustment for chronological age. Given this finding, sex was included as a covariate in relevant subsequent models. There were no significant associations between brain-predicted age and other childhood or demographic factors, including childhood cognitive performance, education level, or socioeconomic status (appendix p 9; p>0·05 in all tests).

**Figure 4:**
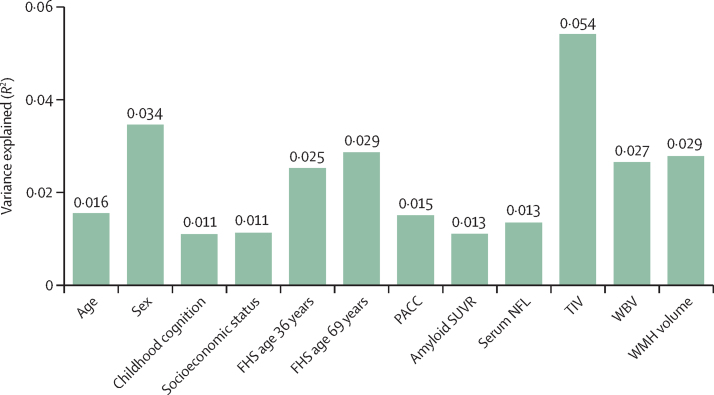
Hierarchical partitioning of significant variables, showing independent contributions of each metric to variance explained in brain-PAD Hierarchical partitioning estimates the percent of variance independently attributable to each metric in a global model in which each is a predictor variable and brain-PAD is the outcome. Combined, these metrics explain *R*^2^=33% of variance in brain-PAD. Brain-PAD=brain predicted age difference. FRS=Framingham risk score. NFL=neurofilament light. PACC=Preclinical Alzheimer's Cognitive Composite Score. SUVR=standardised uptake value ratio. TIV=total intracranial volume. WBV=whole brain volume. WMH=white matter hyperintensity.

The midlife metric of cardiovascular risk was assessed using FRS at age 36 years in 411 participants (appendix p 9), where robust regression showed that, at this age, every 1 SD increase in FRS corresponded with a 2·3-year increase in brain-PAD (95% CI 1·5–3·0; figure 3A). FRS score at age 69 years (443 participants) showed a similar association, with every 1 SD increase in FRS correlating with a 2·6-year older brain-PAD (95% CI 1·9–3·3), despite FRS at age 69 years showing substantially greater variability (SD 13.45) than at age 36 years (SD 1·74; table; Figure 3, Figure 4). Sensitivity analyses showed that these associations remained when whole brain volume was added as a covariate (appendix p 7).

Exploring genetic markers relating to Alzheimer's disease, there was no association between brain-PAD and *APOE* ɛ4 carrier status (456 participants**;** β=0·6 years [95% CI –1·0 to 2·3]) or Alzheimer's disease Polygenic Risk Score (426 participants; β=–0·3 years [–1·0 to 0·5]). Similarly, contemporaneous biomarkers of Alzheimer's disease did not show a significant association with brain-PAD. Although only three participants at the time of study fulfilled criteria for dementia, there was an expected range of fibrillar amyloid β deposition (SUVR) on [^18^F]florbetapir PET scan (appendix p 8); 18% of participants were classified as amyloid β positive.^14^ Neither amyloid deposition nor amyloid status were significantly associated with brain-PAD in this cohort: [^18^F] florbetapir SUVR was associated with β=0·4 years (95% CI –0·3 to 1·1), and amyloid positive status was associated with β=0·6 years (–1·2 to 2·4).

There was evidence of an association between brain-PAD and the blood-based biomarker serum NFL: robust linear regression showed that 1 SD increase in serum NFL was associated with a 1·2-year increase in brain-PAD (95% CI 0·9–1·9; appendix p 8). We observed no differences between participants who were outliers in NFL and the remainder of the cohort, including in rate of major brain disorders and brain volume (appendix p 3).

An older brain-PAD was associated with poorer cognitive performance, with every SD decrease in PACC score being associated with a 1·3-year increase in brain-PAD (95% CI –2·4 to –0·2; figure 3A, appendix p 9). 41 (9%) of 456 participants in the study had a major brain disorder: three with dementia; three with Parkinson's disease; 17 with stroke; ten with other neurological conditions; four with psychiatric disorders; three with a traumatic or neurosurgical condition; and one with a systemic condition (figure 1). These brain-related comorbidities were associated with brain-PAD: the presence of one of these disorders was associated with a 2·8-year increase in brain-PAD (95% CI 0·3 to 5·2; figure 3A, appendix p 9). Although brain-PAD has previously been associated with physical performance—including FEV_1_, grip strength, and walking speed^7^—in 70-year-olds, none of these factors showed an association with brain-PAD (figure 3A; appendix p 9)

Exploring structural imaging metrics, an older brain-PAD was associated with a smaller whole brain volume and a greater WMH burden (figure 3B). There was no association between brain-PAD and ventricular or hippocampal volume.

Using hierarchical partitioning of variance, we explored the independent contribution of 12 metrics—selected on the basis of effect size in univariate analysis—to the variance seen in brain age (figure 4).^24^ Combining these variables in a single linear regression model gave an adjusted *R*^2^ of 0·33 in brain-PAD.

**Figure 5:**
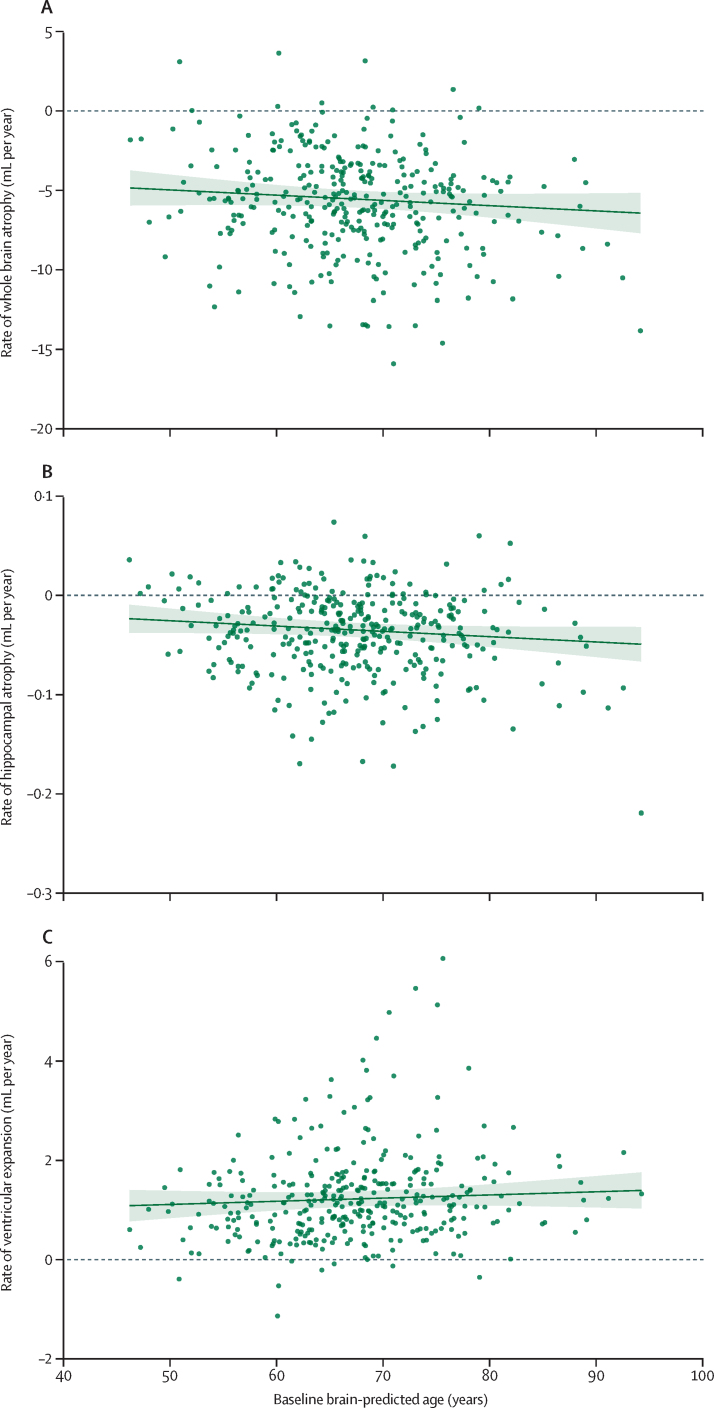
Associations of brain-PAD with brain atrophy rates over the subsequent 2 years Scatter plots show relationship of baseline brain-predicted age with boundary shift integral (mL volume change per year) for whole brain (A), hippocampi (B), and ventricles (C). Scatter plots show the raw data, the green line is the line of best fit from the regression model (adjusted for sex and total intracranial volume), and the shaded area represents 95% CI. Brain-PAD=brain predicted age difference.

In the 345 participants who had an interval scan and did not have dementia, brain-PAD was associated with future rate of hippocampal atrophy: for every 5-year increment in baseline PAD, rates of atrophy increased by 0·003 mL/year (95% CI 0·000 to 0·006; figure 5). This finding was consistent when the model was additionally adjusted for whole brain volume (β=0·003 mL/year per 5-year increment in baseline PAD [0·000 to 0·006]). There was also a directionally consistent relationship between brain PAD and whole brain atrophy rate (0·16 mL/year per 5-year increment in baseline PAD [–0·06 to 0·38]), and ventricular enlargement rate (0·03 mL/year per 5-year increment in baseline PAD [–0·03 to 0·09]).

## Discussion

Using the brain age concept to model biological age, we found that brain-PAD, a single summary metric derived from structural neuroimaging, varies substantially in a narrow age range cohort of older adults. This variability was mechanistically and functionally meaningful, relating to key measures of age-related brain pathology (eg, serum NFL and WMH burden), pre-existing brain diseases, and correlating with cognitive performance. Although brain-PAD was highly correlated with structural brain volume, hierarchical partitioning shows that multiple examined metrics independently contributed to the variance seen in brain-PAD. In addition, brain-PAD was associated with hippocampal atrophy over the following 2-year period. Previous studies have linked brain-PAD with subsequent cognitive decline, dementia, and mortality; however, this is the first study to our knowledge to show the association with brain imaging changes over such a short follow-up period. Although still preliminary, this finding has potential clinical implications—ie, for early identification of people at risk of accelerated ageing, and introduction of early prevention strategies.

Mean brain age was younger than mean chronological age in this study, in keeping with previous observations that Insight 46 participants have relatively better health and cognitive function compared with the wider NSHD cohort. This difference might be partly due to retention bias in the cohort, which has previously been explored,^13^ and due to regression-to-the-mean within the brain age model.^25^ The lower brain age seen in female participants aligns with previous brain age research,^7^ and is compatible with previous studies of this cohort where female participants were found to cognitively outperform male participants.^14^ This difference might also reflect sex differences in life expectancy in the general UK population at age 65 years, where women survive a mean 2·3 years longer than men.

Existing studies have linked socioeconomic status and childhood cognition with both later life cognitive function^26^, ^27^ and WMH burden.^28^, ^29^ Despite these links, brain-PAD was not correlated with prospectively measured childhood assessments in this study, possibly due to the size of the cohort or retention bias in those participants still active in the study. However, the significant association with middle and later life assessments suggests that brain age can capture brain changes that accumulate with ageing; in this case, known lifecourse risk factors for dementia, and imaging features of cerebrovascular pathology. These associations do not extend to the Alzheimer's disease-specific marker of fibrillar amyloid deposition, probably reflecting the largely presymptomatic status of this cohort, and the possibility that the cohort is underpowered to show small to medium size effects. However, the above associations—along with the findings that major brain disorders are associated with brain age—display the utility of brain-PAD as a non-specific metric of a range of brain pathologies. Further variance in brain-PAD might be explained by other pathologies not measured here, including tau, TAR DNA-binding protein 43, and α synuclein.

The relationship between brain-PAD and NFL is notable. NFL is an easily accessible marker of neuroaxonal degeneration, elevated both in the CSF and serum in patients with various neurodegenerative and neurological diseases, and associated with future brain atrophy, mortality, and cognitive decline with longitudinal assessment.^30^, ^31^ NFL also increases with age in healthy individuals. Brain-PAD is an alternative cross-sectional marker that increases with typical and disease-driven ageing, suggesting that common processes might drive changes in both measures. Mechanistically, NFL release is thought to reflect damage to large myelinated axons in the central or peripheral nervous system.^32^ It is likely that common mechanisms might underpin the age-related changes in NFL and brain-predicted age. Avenues for further investigation include more detailed tractography-based analysis of white matter changes, regional brain age analysis focusing on white matter tracts, and corresponding regional gene expression.

This study has several strengths. Participants in the study cohort were recruited at birth during a single week, and are broadly representative of those born in mainland Britain at this time. These participants have been assessed prospectively throughout their lives, allowing robust comparisons of metrics throughout the life course. As has been previously discussed, the generalisability of the study is limited by the cohort consisting entirely of white British participants, reflecting the ethnic homogeneity of the British population in 1946. This homogeneity, along with previously reported recruitment and retention biases in Insight 46 (eg, higher educational attainment, non-manual socioeconomic position, and better self-rated health),^33^ limit generalisability, especially for early life metrics, which will be most affected by cohort attrition. Retention bias might also account for the reason the model was not able to replicate associations with physical health metrics, including grip strength, walking speed, and lung function, which have been seen in a different cohort using a similar brain age model.^7^ Replication in more diverse populations and in cohorts of different ages is required before the findings can be confirmed. The short follow-up period between scans might also limit power for the atrophy-related metrics, as typical age-related volume decrease might be subtle. Comparisons across analyses were limited by data availability, leading to inconsistent sample sizes in the various models. As we aimed to explore the multiple potential contributions to brain ageing, rather than the factors showing the most influence, we chose not to correct for multiple comparisons, which would probably increase type II errors. Although the chosen approach might increase the number of type 1 errors, we opted to use it to identify potential avenues for future research.^34^ The current brain age model uses T1-weighted MRI, so only reflects variability in brain structure and volume, and is not driven by patterns of WMHs, iron deposition, or axonal degeneration. Alternative brain ages using T2-weighted or diffusion-weighted MRI are available,^35^, ^36^ although T1-weighted MRI has consistently shown very accurate age prediction and is highly reliable.^37^ Moreover, since T1-weighted MRI has been validated far more extensively,^9^ our results can be readily compared with most of the brain age literature. In this study, brain age was assessed at a single timepoint to reflect how it might be used clinically: as a cross-sectional measure indexing multiple aspects of brain health into a summary metric. A further longitudinal study following changes in brain age would be of interest and will be the subject of future work.

We have shown that brain-PAD relates to both general and disease-specific contributions to age-related brain changes, including multiple brain imaging metrics and life course metrics. Further exploration entails longitudinal follow-up, which is currently underway with phase 3 of the Insight 46 study, with more detailed cognitive, imaging, and biomarker assessment. Additionally, 30% of participants have consented to post-mortem brain examinations. Crucially, these longitudinal assessments will allow further exploration of the brain age concept and its potential use as a means of integrating the effects of a range of pathologies and predicting future decline.

## Data sharing

Anonymised data will be shared by request from qualified investigators from the Medical Research Council National Survey for Health and Development. Please contact the corresponding author for data sharing purposes. Details of R packages and analysis code is available online at GitHub.

## Declaration of interests

This research was funded by a Wolfson Clinical Research Fellowship awarded to AZW, and a Selfridges Group Foundation award (UB170045), with leveraged funding from Alzheimer's Research UK (ARUK-PG2014-1946, ARUK-PG2017-1946), Medical Research Council Dementia Platforms UK (CSUB19166), the Wolfson Foundation (PR/ylr/18575) and the Alzheimer's Association (SG-666374-UK BIRTH COHORT). AZW has served as a medical monitor for Neuroscience Trials Australia receiving no personal compensation, and has an Alzhiemer's Research UK travel grant. CAL is now a full-time employee of Roche Products and a shareholder in Hoffmann La Roche. HZ has served on scientific advisory boards and as a consultant for AbbVie, Alector, ALZPath, Annexon, Apellis, Artery Therapeutics, AZTherapies, CogRx, Denali, Eisai, Nervgen, Novo Nordisk, Pinteon Therapeutics, Red Abbey Labs, reMYND, Passage Bio, Roche, Samumed, Siemens Healthineers, Triplet Therapeutics, and Wave, has given lectures in symposia sponsored by Cellectricon, Fujirebio, Alzecure, Biogen, and Roche, and is a co-founder of Brain Biomarker Solutions in Gothenburg, which is a part of the GU Ventures Incubator Program (outside the submitted work). HZ is a Wallenberg Scholar supported by grants from the Swedish Research Council (2018- 02532), the European Research Council (681712), Swedish State Support for Clinical Research (ALFGBG-720931), the Alzheimer Drug Discovery Foundation (USA [201809- 2016862]), the Alzheimer's Disease Strategic Fund and the Alzheimer's Association (ADSF-21–831376-C, ADSF-21–831381-C, and ADSF-21–831377-C), the Olav Thon Foundation, the Erling-Persson Family Foundation, Stiftelsen för Gamla Tjänarinnor, Hjärnfonden, Sweden (FO2019–0228), the EU's Horizon 2020 research and innovation programme under the Marie Skłodowska-Curie (grant agreement number 860197 [MIRIADE]), the EU Joint Program for Neurodegenerative Disorders (JPND2021–00694), and the UK Dementia Research Institute at UCL. HZ is the Chair of the Alzheimer's Associaiton Global Biomarker Standardization Consotrium. FB is on the steering committee or is an iDMC member for Biogen, Merck, Roche, EISAI, and Prothena, and is a consultant for Roche, Biogen, Merck, IXICO, Jansen, and Combinostics. FB has research agreements with Merck, Biogen, GE Healthcare, and Roche, is a co-founder and shareholder of Queen Square Analytics, and a board member of the journals *Neurology, Radiology, MSJ, and Neuroradiology*. He was Editor In Chief of Clinical Neuroradiology – the ESNR textbook (Springer), and has had projects funced by the UK MS Society, Dutch Foundation MS Research, NOW (Picture project) and IMI-EU (Amypad project). NCF's research group has received payment for consultancy or for conducting studies from Biogen, Eli Lilly Research Laboratories, Ionis, and Roche. NCF receives no personal compensation for the aforementioned activities. NCF has served on a Data Safety Monitoring Board for Biogen. JHC is a scientific consultant for Claritas HealthTech and Queen Square Analytics, and a shareholder in Claritas HealthTech. JMS has received research funding from Avid Radiopharmaceuticals (a wholly owned subsidiary of Eli Lilly), has consulted for Roche Pharmaceuticals, Biogen, Merck, and Eli Lilly, has given educational lectures sponsored by GE Healthcare, Eli Lilly, and Biogen, and serves on a Data Safety Monitoring Committee for Axon Neuroscience SE. The genetic analyses were funded by the Brain Research Trust (UCC14191). Avid Radiopharmaceuticals, a wholly owned subsidiary of Eli Lilly, kindly provided the ^18^F-florbetapir tracer free of cost, but had no role in the design, conduct, analysis, or reporting of Insight 46 study findings. AK was supported by a Wolfson Clinical Research Fellowship and a Weston Brain Institute and Selfridges Group Foundation award (UB170045) that also funded the serum NFL analyses. The HD-1 Analyser at UCL was funded by a Multi-User Equipment grant from the Wellcome Trust. TDP was supported by a Wellcome Trust Clinical Research Fellowship (200109/Z/15/Z) and is supported by a National Institute for Health and Care Research clinical lectureship. The NSHD, MR, and AW are funded by the MRC (MC_UU_00019/1, MC_UU_00019/3), with MR acknowledging additional support from the US Alzheimer's Society. VE-P would like to thank the Joint Programming for Neurodegeneration (JPND—MRC: MR/T04604X/1) and the Medical Research Council (MRC) Centre for Neuropsychiatric Genetics and Genomics (MRC: MR/L010305/1). FB, NCF, and JMS are supported by the National Institute for Health Research Queen Square Dementia Biomedical Research Unit and the Leonard Wolfson Experimental Neurology Centre. GL was supported by the UK Dementia Research Institute at Cardiff, UK. JMS is supported by UCL Hospitals Biomedical Research Centre, Engineering and Physical Sciences Research Council (EP/J020990/1), British Heart Foundation (PG/17/90/33415), and the EU's Horizon 2020 research and innovation programme (666992). JHC acknowledges funding from UK Research and Innovation and the MRC (MR/R024790/2). All other authors declare no competing interests.
