# Supplementary material for: Life course, genetic, and neuropathological associations with brain age in the 1946 British Birth Cohort: a population-based study
Source: Lancet Healthy Longev. 2022 Sep;3(9):e607–16. doi: 10.1016/S2666-7568(22)00167-2 (PMC10499760; doi:10.1016/S2666-7568(22)00167-2)

THE LANCET
Healthy Longevity

## Supplementary appendix

This appendix formed part of the original submission and has been peer reviewed. We post it as supplied by the authors.

Supplement to: Wagen AZ, Coath W, Keshavan A, et al. Life course, genetic, and neuropathological associations with brain age in the 1946 British Birth Cohort: a population-based study. *Lancet Healthy Longev* 2022; published online Aug 22. https://doi.org/10.1016/S2666-7568(22)00167-2.

## Contents:

Supplementary Note 1 p1
Supplementary Table 1 p4
Supplementary Table 2 p6
Supplementary Table 3 p7
Supplementary Table 4 p8
Supplementary Figure 1 p9
Supplementary Figure 2 p10

# Life course, genetic and neuropathological associations with brain age in the British 1946 Birth Cohort: a population-based study

## Supplementary Appendix

**Supplementary Note 1.** Detailed methods for analysis of brain imaging data, derivation of AD polygenic risk score, and blood-based biomarkers.

Participants were scanned on a single Biograph mMR 3T PET/MRI scanner (Siemens Healthcare, Erlangen Germany) after injection of 370MBq of the Aβ- PET ligand $^{18}$F-florbetapir (Amyvid), simultaneously acquiring dynamic PET and multimodal MRI data.
PET data were processed using an automated in-house processing pipeline, including pseudo-computed tomography attenuation correction, as has been described previously.[1] Amyloid burden was assessed using standardised uptake value ratio (SUVR) from static images acquired 50 to 60 minutes post-injection. A global measure was calculated from a composite of cortical regions of interest - including the lateral and medial frontal, anterior and posterior cingulate, lateral parietal and lateral temporal regions – with a reference region of eroded subcortical white matter and Iterative Yang partial volume correction applied. Amyloid-positive status was determined by a Gaussian mixed model applied to SUVR values, with a cut-point of 0·671 or 17 centiloids[2], which corresponded to the 99$^{th}$ percentile of the lower (amyloid-negative) Gaussian curve.
MR acquisitions used a body coil RF transmitter in conjunction with a 12-channel receiver array head coil, with a maximum gradient strength of 45mT/m along each direction. High resolution three-dimensional (3D) volumetric T1-weighted and FLAIR MRI images were obtained using a magnetisation prepared rapid gradient echo (MPRAGE) sequence, and long echo train turbo spin echo (SPACE) sequence respectively.[3,4] Images were reviewed by a radiologist to assess for any major brain disorders.[5] Images underwent manual quality control (QC) and pre-processing including correction for gradient non-linearity and brain masked N4-bias correction.[6,7] They then underwent automated segmentation, with manual editing where required, to derive whole brain volume (WBV) via Multi-Atlas Propagation and Segmentation, and hippocampal volume (HV) via Similarity and Truth Estimation for Propagated Segmentations.[8,9] Ventricular volumes were derived manually via thresholding, with no values included greater than 60% mean whole brain intensity. Statistical parametric mapping software (SPM12; Wellcome Centre for Human Neuroimaging) was used to derive total intracranial volume (TIV).[10] WMH burden including subcortical grey matter but excluding infratentorial regions was derived from T1/FLAIR images using a Bayesian model selection (BaMoS) algorithm with visual quality control. Manual editing was necessary in 29 cases, 17 of which were due to misclassified flow artefact, 12 due choroid plexus missegmentation, and 8 due to cortical stroke misclassification. This was performed by a trained rater, whose inter-rater $R^2$ with another expert tested on 30 scans was 0·999 and Dice score was 0·94.[11,12] Changes in whole brain, ventricular and hippocampal volume were calculated from baseline and repeat MRI using the boundary shift integral (BSI).[13] Specifically, the k-means normalised BSI was used to calculate whole brain atrophy following affine registration of scan pairs and differential bias correction (DBC)[14]. Ventricular expansion was determined using affine whole-brain registration, followed by an additional rigid registration using the ventricle regions only, and calculation of BSI without DBC. Hippocampal atrophy was assessed using affine whole-brain registration, followed by an additional rigid registration focusing on the hippocampus and surrounding regions, with DBC and calculation of BSI using a double intensity window approach.[15] Total hippocampal BSI was calculated as the sum of left and right.

To derive the Alzheimer's Disease polygenic risk score, genomic data were processed from 2864 individuals in the NSHD, including 2835 samples using 486,137 SNPs from the NeuroX2 platform and 2851 samples using 476,728 SNPs from the DrugDev platform. Quality-control (QC) analysis was performed using

PLINK2 (www.cog-genomics.org/plink/2·0/).[16] Samples were merged on 637,216 common SNPs (where 2822 individuals had overlapping genetic information), with 473,381 autosomal SNPs remaining after the initial QC. Those SNPs were further imputed using the Haplotype Reference Consortium v1·1 panel on Michigan Imputation Server.[17] Variant call format files were converted into standard plink format and final QC filtering was applied. SNPs were excluded based on the following criteria: imputation information metric (INFO) scores <0·7; imputation posterior probability <0·9; SNPs that were not biallelic; minor allele frequency <0·05; call rate <95%; or Hardy Weinberg Equilibrium P<1e-6. Participants were excluded with call rates <95%; heterozygosity (HET > |0·05|); relatedness based on proportion of identity by descent (PI-HAT) <0·2 and Principal Component Analysis (PCA). The NSHD cohort was merged with the 1000G dataset (http://www.1000genomes.org/) and PCA outliers that did not cluster near European individuals were removed. Finally, 2729 individuals on 4,145,518 markers were retained for the further analysis.
PRS were computed in PLINK using a standard method of pruning and thresholding (P+T).[18] The summary statistics from the latest available clinically assessed case-control GWAS on AD were used (stage 1)[19] to generate genetic scores. For this study we used linkage disequilibrium-pruned markers with $r^2 > 0{\cdot}1$ in 1000-kb window and *P* value cut-off of 0·5 which was found to be optimal choice from previous work.[20] All PRS were adjusted for 8 principle components and standardised.

Non-fasted serum samples for blood-based biomarker detection were collected from a peripheral vein into 8·5 ml SST tubes. These were transported to the laboratory at room temperature within 30 minutes and centrifuged at 2000g at room temperature for 10 minutes. Five hundred µL aliquots in polypropylene cryovials were stored at -80ºC until all samples had been collected. For serum NFL analysis, a single 500 µL aliquot of serum for each individual was thawed directly to room temperature over 1 hour and vortexed to ensure thorough mixing. Two hundred µL was pipetted into a 1·5 ml polypropylene centrifuge tube and centrifuged at 13 000 g for 10 minutes, as per the kit manufacturer's recommendation; the remaining 300 µL was replaced into -80 ºC in the original cryovial. After the 200 µL was centrifuged, 130 µL of the supernatant was pipetted onto the plate for analysis in duplicate, using commercially available Simoa immunoassay NF-Light kits of the same batch (Quanterix, Billerica, MA). If the coefficient of variation (CV) across the duplicates was >15% or no value was returned for either, the procedure above was repeated at a later date, employing one additional freeze-thaw cycle by starting with the 300 µL volume that was in the original cryovial. The decision to allow two freeze thaw cycles on samples for NFL quantification was justified by prior work thatshows that this analyte is stable for up to four freeze-thaw cycles.[21] By this method, 428 samples underwent one freeze-thaw cycle and 72 samples underwent two freeze-thaw cycles. All 500 individuals who had blood sampling had a serum NFL value quantified with an intra-assay CV <15%, and inter-assay CV for two run validation controls were both 11%.

References


1 Burgos N, Cardoso MJ, Thielemans K, *et al.* Multi-contrast attenuation map synthesis for PET/MR scanners: assessment on FDG and Florbetapir PET tracers. *Eur J Nucl Med Mol Imaging* 2015; **42**: 1447–58.
2 Coath W, Modat M, Cardoso J, *et al.* IC-P-007: CENTILOID SCALE TRANSFORMATION OF FLORBETAPIR DATA ACQUIRED ON A PET/MR SCANNER. *Alzheimer's Dement* 2019; **15**: P17–8.
3 Mugler JP 3rd, Brookeman JR. Rapid three-dimensional T1-weighted MR imaging with the MP-RAGE sequence. *J Magn Reson Imaging* 1991; **1**: 561–7.
4 Mugler III JP. Optimized three-dimensional fast-spin-echo MRI. *J Magn Reson Imaging* 2014; **39**: 745–67.
5 Keuss SE, Parker TD, Lane CA, *et al.* Incidental findings on brain imaging and blood tests: Results from the first phase of Insight 46, a prospective observational substudy of the 1946 British birth cohort. *BMJ Open* 2019; **9**: 1–8.
6 Jovicich J, Czanner S, Greve D, *et al.* Reliability in multi-site structural MRI studies: Effects of gradient non-linearity correction on phantom and human data. *Neuroimage* 2006; **30**: 436–43.
7 Tustison NJ, Avants BB, Cook PA, *et al.* N4ITK: improved N3 bias correction. *IEEE Trans Med Imaging* 2010; **29**: 1310—1320.
8 Leung KK, Barnes J, Modat M, *et al.* Brain MAPS: An automated, accurate and robust brain extraction technique using a template library. *Neuroimage* 2011; **55**: 1091–108.
9 Jorge Cardoso M, Leung K, Modat M, *et al.* STEPS: Similarity and Truth Estimation for Propagated Segmentations and its application to hippocampal segmentation and brain parcelation. *Med Image Anal* 2013; **17**: 671–84.
10 Malone IB, Leung KK, Clegg S, *et al.* Accurate automatic estimation of total intracranial volume: A

nuisance variable with less nuisance. *Neuroimage* 2015; **104**: 366–72.
11 Sudre CH, Cardoso MJ, Bouvy WH, Biessels GJ, Barnes J, Ourselin S. Bayesian Model Selection for Pathological Neuroimaging Data Applied to White Matter Lesion Segmentation. *IEEE Trans Med Imaging* 2015; **34**: 2079–102.
12 Lane CAS. The influence of life course vascular risk on brain pathologies and cognition in later life- a neuroimaging study of the British 1946 birth cohort. 2018. https://discovery.ucl.ac.uk/id/eprint/10067233/1/Lane_RPSDOWN_thesis.pdf (accessed Sept 26, 2021).
13 Freeborough PA, Fox NC. The boundary shift integral: An accurate and robust measure of cerebral volume changes from registered repeat MRI. *IEEE Trans Med Imaging* 1997; **16**: 623–9.
14 Leung KK, Clarkson MJ, Bartlett JW, *et al.* Robust atrophy rate measurement in Alzheimer's disease using multi-site serial MRI: Tissue-specific intensity normalization and parameter selection. *Neuroimage* 2010; **50**: 516–23.
15 Leung KK, Barnes J, Ridgway GR, *et al.* Automated cross-sectional and longitudinal hippocampal volume measurement in mild cognitive impairment and Alzheimer's disease. *Neuroimage* 2010; **51**: 1345–59.
16 Chang CC, Chow CC, Tellier LCAM, Vattikuti S, Purcell SM, Lee JJ. Second-generation PLINK: Rising to the challenge of larger and richer datasets. *Gigascience* 2015; **4**: 1–16.
17 Das S, Forer L, Schönherr S, *et al.* Next-generation genotype imputation service and methods. *Nat Genet* 2016; **48**: 1284–7.
18 Purcell SM, Wray NR, Stone JL, *et al.* Common polygenic variation contributes to risk of schizophrenia and bipolar disorder. *Nature* 2009; **460**: 748–52.
19 Kunkle BW, Grenier-Boley B, Sims R, *et al.* Genetic meta-analysis of diagnosed Alzheimer's disease identifies new risk loci and implicates Aβ, tau, immunity and lipid processing. *Nat Genet* 2019; **51**: 414–30.
20 Escott-Price V, Sims R, Bannister C, *et al.* Common polygenic variation enhances risk prediction for Alzheimer's disease. *Brain* 2015; **138**: 3673–84.
21 Keshavan A, Heslegrave A, Zetterberg H, Schott JM. Stability of blood-based biomarkers of Alzheimer's disease over multiple freeze-thaw cycles. *Alzheimer's Dement Diagnosis, Assess Dis Monit* 2018; **10**: 448–51.

**Supplementary Table 1**. Summary statistics comparing those participants included in this study, those excluded due to missing data, and the outliers from the serum NFL analysis as defined by Tukey's Fences.

| Summary Statistics | Sample used in study | Sample excluded from study | sNFL outliers |
|---|---|---|---|
| **Chronological age (years)** | | | |
| Number | 456 | 46 | 14 |
| Range | 69·25, 71·87 | 69·31, 71·69 | 69·57, 71·85 |
| Mean (SD) | 70·67 (0·67) | 70·70 (0·69) | 70·87 (0·79) |
| **Brain predicted age (years)** | | | |
| Number | 456 | 12 | 14 |
| Range | 46·29, 94·26 | 55·67, 82·23 | 55·23, 91·16 |
| Mean (SD) | 67·90 (8·15) | 70·51 (8·46) | 74·08 (9·74) |
| **Brain predicted age difference (years)** | | | |
| Number | 456 | 12 | 14 |
| Range | -24·59, 22·70 | -15·07, 11·10 | -16·62, 20·61 |
| Mean (SD) | -2·77 (8·04) | -0·14 (8·22) | 3·21 (9·77) |
| **Sex** | | | |
| Female | 225 (49%) | 21 (46%) | 5 (36%) |
| **Socioeconomic status** | | | |
| Manual | 70 (15%) | 6 (13%) | 2 (14%) |
| Non-manual | 386 (85%) | 40 (87%) | 12 (86%) |
| **Educational attainment** | | | |
| None attempted | 70 (15%) | 8 (17%) | 3 (21%) |
| School grade | 139 (30%) | 12 (26%) | 5 (36%) |
| Higher education | 247 (54%) | 26 (57%) | 6 (43%) |
| **Childhood cognition (z-score)** | | | |
| Number | 456 | 46 | 14 |
| Range | -1·60, 2·50 | -1·31, 1·53 | -1·31, 1·25 |
| Mean (SD) | 0·41 (0·75) | 0·23 (0·68) | 0·05 (0·76) |
| **Smoking** | | | |
| Never smoked | 160 (35%) | 11 (24%) | 4 (29%) |
| Ex-smoker | 280 (61%) | 33 (72%) | 10 (71%) |
| Current smoker | 16 (4%) | 2 (4%) | 0 (0%) |
| **Major brain disorder** | | | |
| None | 415 (91%) | 45 (98%) | 12 (86%) |
| Present | 41 (9%) | 1 (2%) | 2 (14%) |
| **Total intracranial volume (ml)** | | | |
| Number | 456 | 12 | 14 |
| Range | 1,114, 1,939 | 1,358, 1,746 | 1,289, 1,600 |
| Mean (SD) | 1,431 (133) | 1,521 (112) | 1,448 (95) |
| **Whole brain volume (ml)** | | | |
| Number | 456 | 12 | 14 |
| Range | 819, 1,494 | 969, 1,305 | 969, 1,263 |
| Mean (SD) | 1,099 (99) | 1,156 (96) | 1,109 (101) |
| **Ventricular volume (ml)** | | | |
| Number | 456 | 12 | 14 |
| Range | 6·16, 112·00 | 20·03, 65·48 | 18·43, 85·33 |
| Mean (SD) | 30·94 (16·34) | 37·14 (15·55) | 39·98 (20·85) |
| **Hippocampal volume (ml)** | | | |
| Number | 456 | 12 | 14 |
| Range | 4·12, 8·54 | 4·92, 7·45 | 4·51, 7·81 |
| Mean (SD) | 6·26 (0·67) | 6·30 (0·77) | 6·10 (0·96) |
| **White matter hyperintensity volume (ml)** | | | |

| Summary Statistics | Sample used in study | Sample excluded from study | sNFL outliers |
|---|---|---|---|
| Number | 456 | 14 | 14 |
| Range | 0·27, 33·67 | 0·38, 15·99 | 1·22, 24·90 |
| Mean (SD) | 5·21 (5·54) | 5·12 (4·14) | 6·95 (7·15) |
| **PACC score (z-score)** | | | |
| Number | 456 | 46 | 14 |
| Range | -3·49, 1·72 | -1·54, 1·59 | -3·48, 1·58 |
| Mean (SD) | -0·01 (0·74) | 0·05 (0·70) | -0·69 (1·30) |
| **Amyloid status** | | | |
| Negative | 373 (82%) | 5 (83%) | 10 (71%) |
| Positive | 83 (18%) | 1 (17%) | 4 (29%) |
| **Amyloid beta SUVR (centiloids)** | | | |
| Number | 456 | 6 | 14 |
| Range | -17·94, 92·84 | -9·38, 21·20 | -0·82, 54·96 |
| Mean (SD) | 7·13 (19·05) | 2·32 (11·59) | 18·96 (18·72) |
| **Serum Neurofilament light (pg/ml)** | | | |
| Number | 456 | 44 | 14 |
| Range | 7·26, 124·00 | 7·21, 46·90 | 38·60, 124·00 |
| Mean (SD) | 20·74 (12·19) | 20·63 (7·91) | 69·17 (34·03) |
| **APOE ε4 status** | | | |
| Non-carrier | 325 (71%) | 27 (61%) | 10 (71%) |
| Carrier | 131 (29%) | 17 (39%) | 4 (29%) |
| **Alzheimer's Polygenic Risk Score (z-score)** | | | |
| Number | 426 | 44 | 13 |
| Range | -3·15, 2·75 | -1·96, 1·30 | -1·55, 2·75 |
| Mean (SD) | -0·06 (1·01) | -0·30 (0·89) | -0·21 (1·15) |
| **Framingham Risk Score age 36** | | | |
| Number | 411 | 44 | 12 |
| Range | 0·58, 11·25 | 0·88, 9·08 | 1·57, 6·91 |
| Mean (SD) | 2·90 (1·74) | 3·14 (2·09) | 3·56 (1·82) |
| **Framingham Risk Score age 69** | | | |
| Number | 443 | 45 | 13 |
| Range | 2·53, 68·75 | 7·89, 83·87 | 11·67, 50·97 |
| Mean (SD) | 25·90 (13·45) | 28·77 (16·17) | 27·97 (10·58) |
| **FEV1 (L)** | | | |
| Number | 409 | 41 | 14 |
| Range | 0·37, 4·84 | 1·43, 4·06 | 0·84, 4·20 |
| Mean (SD) | 2·71 (0·68) | 2·80 (0·69) | 2·73 (0·82) |
| **Grip strength (kg)** | | | |
| Number | 449 | 45 | 14 |
| Range | 11·00, 61·50 | 16·80, 57·40 | 17·20, 58·60 |
| Mean (SD) | 32·99 (10·90) | 34·96 (11·21) | 35·54 (13·42) |
| **Walking speed (m/s)** | | | |
| Number | 431 | 44 | 13 |
| Range | 0·57, 2·22 | 0·27, 1·78 | 0·67, 1·84 |
| Mean (SD) | 1·08 (0·26) | 1·06 (0·28) | 1·00 (0·29) |

**Supplementary Table 2.** Global model showing overall variance explained by included variables.

LINEAR REGRESSION RESULTS
*Observations:* N = 325
*Dependent Variable:* PAD

MODEL FIT:
$F(24,300) = 7{\cdot}25$, $p = 0{\cdot}00$
$R^2 = 0{\cdot}37$
*Adj.* $R^2 = 0{\cdot}32$

*Standard errors: OLS*

| | **Estimate** | **2·5%** | **97·5%** | **P** |
|---|---|---|---|---|
| (Intercept) | -1·34 | -5·0768 | 2·39 | 4·8e-01 |
| Age (z-score) | 0·79 | 0·0069 | 1·58 | 4·8e-02 |
| Sex - male | -0·87 | -4·5354 | 2·80 | 6·4e-01 |
| Childhood cognition | 0·45 | -0·7746 | 1·68 | 4·7e-01 |
| Education: Vocational or GCSE (reference: none) | -1·57 | -4·1521 | 1·01 | 2·3e-01 |
| Education: A-level or higher (reference: none) | -1·34 | -4·0471 | 1·36 | 3·3e-01 |
| Socioeconomic status: Non-manual (reference: manual) | 0·86 | -1·5935 | 3·32 | 4·9e-01 |
| Smoking: Ex-smoker (reference: non smoker) | -1·29 | -2·9659 | 0·38 | 1·3e-01 |
| Smoking: Current smoker (reference: non smoker) | -0·85 | -5·7528 | 4·05 | 7·3e-01 |
| Total intracranial volume (z-score) | 6·81 | 4·5157 | 9·11 | 1·4e-08 |
| Whole brain volume (z-score) | -4·38 | -6·3471 | -2·41 | 1·6e-05 |
| Ventricular volume (z-score) | -0·58 | -1·6061 | 0·44 | 2·6e-01 |
| Hippocampal volume (z-score) | 1·04 | 0·0642 | 2·01 | 3·7e-02 |
| WMH Volume (z-score) | 1·91 | 1·1283 | 2·68 | 2·3e-06 |
| PACC | -0·61 | -1·8763 | 0·66 | 3·5e-01 |
| Amyloid beta centioids (z-score) | 0·23 | -0·6516 | 1·11 | 6·1e-01 |
| ApoE4 Carrier (reference: non carrier) | 0·16 | -1·6900 | 2·00 | 8·7e-01 |
| AD Polygenic risk score | -0·36 | -1·0956 | 0·38 | 3·4e-01 |
| Serum NFL (z-score) | 1·41 | 0·6578 | 2·16 | 2·7e-04 |
| FRS age 36 (z-score) | 0·50 | -0·6161 | 1·61 | 3·8e-01 |
| FRS age 69 (z-score) | 0·62 | -0·5483 | 1·79 | 3·0e-01 |
| FEV1 (z-score) | 0·15 | -1·0828 | 1·38 | 8·1e-01 |
| Grip strength (z-score) | -0·19 | -1·4293 | 1·04 | 7·6e-01 |
| Walking speed (z-score) | -0·25 | -1·0469 | 0·54 | 5·3e-01 |
| Height (z-score) | 0·23 | -1·2049 | 1·66 | 7·5e-01 |

**Supplementary Table 3.** Sensitivity analysis of Framingham risk score models at age 36 and contemporary, additionally covarying for whole brain volume. Results show beta estimate, the lower (2·5%) and higher (97·5%) confidence intervals, and the P value.

LINEAR REGRESSION RESULTS: FRAMINGHAM RISK AGE 36
*Observations:* 411
*Dependent Variable:* PAD

MODEL FIT:
$F(5,437) = 12{\cdot}5392$, $p = 0{\cdot}0000$
$R^2 = 0{\cdot}106$
*Adj.* $R^2 = 0{\cdot}1155$

| | **Estimate** | **2·5%** | **97·5%** | **P** |
|---|---|---|---|---|
| Intercept | -3·9 | -5·65 | -2·1 | 2·9e-05 |
| Framingham risk score at age 36 | 1·9 | 1·06 | 2·7 | 7·4e-06 |
| Non-manual socioeconomic Status | 1·4 | -0·63 | 3·3 | 1·8e-01 |
| Whole brain volume (ml) | 1·4 | 0·50 | 2·2 | 1·9e-03 |

LINEAR REGRESSION RESULTS: FRAMINGHAM RISK AGE 69
*Observations:* 443
*Dependent Variable:* PAD

MODEL FIT:
$F(3,439) = 24{\cdot}6741$, $p = 0{\cdot}0000$
$R^2 = 0{\cdot}1443$
*Adj.* $R^2 = 0{\cdot}1384$

| | **Estimate** | **2·5%** | **97·5%** | **P** |
|---|---|---|---|---|
| Intercept | -4·2 | -5·97 | -2·3 | 8·5e-06 |
| Framingham risk score at age 69 | 2·2 | 1·50 | 2·9 | 3·0e-09 |
| Non-manual socioeconomic Status | 1·6 | -0·37 | 3·6 | 1·1e-01 |
| Whole brain volume (ml) | 1·5 | 0·75 | 2·2 | 7·3e-05 |

**Supplementary Table 4.** Metrics utilised in regression models

| **Model** | **Model type** | **Metrics utilised** |
|---|---|---|
| Age | Linear regression | PAD ~ Age |
| Sex | Linear regression | PAD ~ Sex + Age |
| Educational attainment | Linear regression | PAD ~ Education + Sex + Age |
| Socioeconomic status | Linear regression | PAD ~ SocioStatus + Sex + Age |
| Childhood cognition | Linear regression | PAD ~ Childcog + Sex + Age |
| Framingham risk age 36 | Robust regression | PAD ~ FHS_36z + SocioStatus |
| Framingham risk age | Linear regression | PAD ~ FHS_69z + SocioStatus |
| Serum NFL | Robust regression | PAD ~ SerumNFLz + Sex + Age |
| Amyloid SUVR | Linear regression | PAD ~ AmyloidSuvrz + Sex + Age |
| Amyloid status | Linear regression | PAD ~ AmyloidStatus + Sex + Age |
| APOE4 status | Linear regression | PAD ~ ApoE4 |
| AD Polygenic risk score | Linear regression | PAD ~ PRS |
| Cognition (PACC) | Linear regression | PAD ~ PACC + Sex + Education + Childcog + SocioStatus + Age |
| Major brain disorders | Linear regression | PAD ~ MajorBrainDisorder + Age + Sex |
| FEV1 | Linear regression | PAD ~ FEV1z + Smoking + Heightz + Age + Sex |
| Grip strength | Linear regression | PAD ~ GripStrengthz + Sex + Age |
| Walking speed | Linear regression | PAD ~ WalkSpeedz + Heightz + Sex + Age |
| Whole brain volume | Linear regression | PAD ~ WBVz + TIVz + Sex + Age |
| Hippocampal volume | Linear regression | PAD ~ Hippovolz + Sex + TIVz + Age |
| Ventricular volume | Linear regression | PAD ~ Ventvolz + TIVz + Sex + Age |
| WMH volume | Linear regression | PAD ~ WMHVolz + TIVz + Sex + Age |
| Prediction of whole brain volume change | Linear regression | BrainBSI ~ ScanInterval + PADadjust:ScanInterval + Sex:ScanInterval + TIVz:ScanInterval + 0 |
| Prediction of hippocampal volume change | Robust regression | (HippoBSI ~ ScanInterval + PADadjust:ScanInterval + Sex:ScanInterval + TIVz:ScanInterval + 0 |
| Prediction of ventricular volume change | Linear regression | VentBSI ~ ScanInterval + PADadjust:ScanInterval + Sex:ScanInterval + TIVz:ScanInterval + 0 |

**Supplementary Figure 1.** Correlations of brain predicted age with variables of interest: Framingham risk score at age 36 (A) and 69 (B); serum NFL (C); amyloid SUVR (D); PACC (E); major brain disorder (F); whole brain volume (G); and white matter hyperintensities (H).
Abbreviations: NFL, neurofilament light; SUVR, standardised uptake value ratio; PACC, preclinical Alzheimer's cognitive composite.

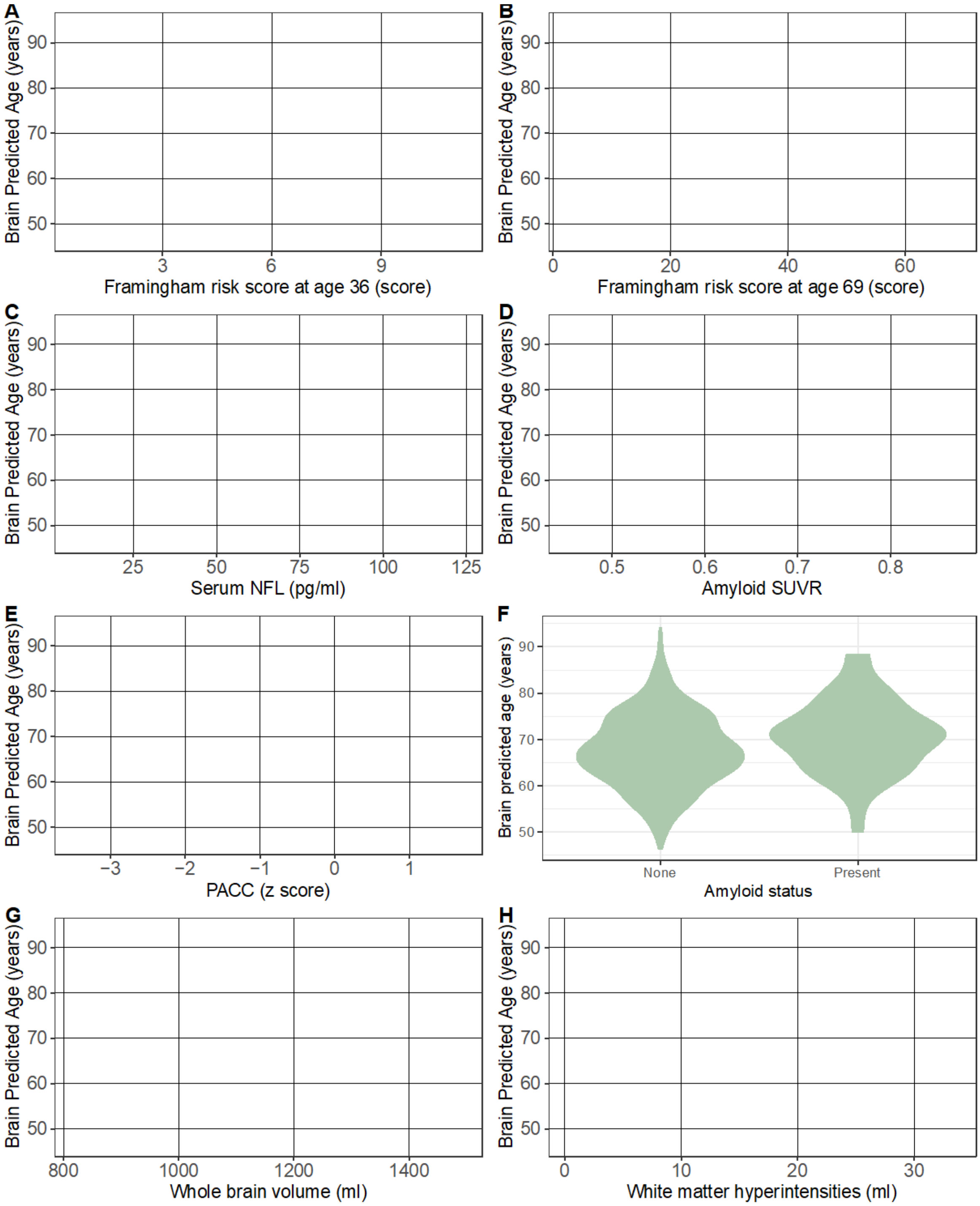

**Supplementary Figure 2.** Sensitivity analyses of regression results with age added as a covariate to relevant linear models. Abbreviations: FRS, Framingham risk score; NFL, neurofilament light; SUVR, standardised uptake value ratio; PACC, preclinical Alzheimer's cognitive composite; WBV, whole brain volume; WMH, white matter hyperintensity; brain-PAD, brain predicted age difference.

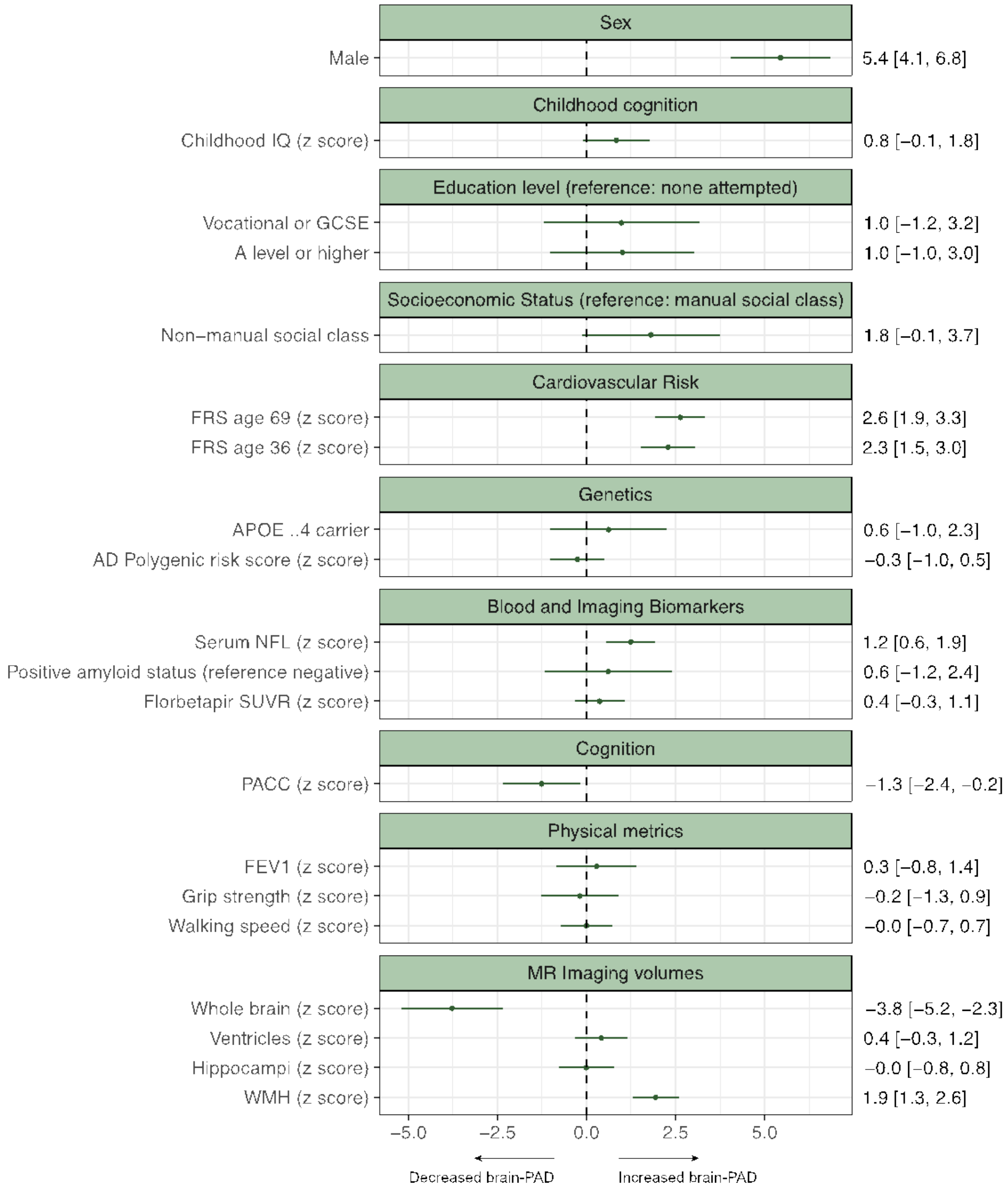

Supplement: Supplementary appendix [file mmc1.pdf]
